# Angiogenin-loaded fibrin/bone powder composite scaffold for vascularized bone regeneration

**DOI:** 10.1186/s40824-015-0040-4

**Published:** 2015-08-25

**Authors:** Beom-Su Kim, Jin-Seong Kim, Sun-Sik Yang, Hyung-Woo Kim, Hun Jun Lim, Jun Lee

**Affiliations:** Wonkwang Bone Regeneration Research Institute, Wonkwang University, Iksan, 570-749 Korea; Bonecell Biotech Inc., Dunsan-dong, Seo-gu, Daejeon, 302-830 Korea; Department of Herbal Crop Research, NIHHS, RDA, Eumseong, 369-873 Korea; Department of Dentistry, Oral and Maxillofacial, Wonkwang University, Iksan, 570-749 Korea

**Keywords:** Angiogenin, Fibrin, Scaffold, Bone regeneration, Angiogenesis

## Abstract

**Background:**

Angiogenin (ANG) is a potent stimulator of angiogenesis. The aim of this study was to fabricate an ANG-loaded scaffold and to evaluate its angiogenic and osteogenic effects. In this study, we fabricated an ANG-loaded scaffold using bovine bone powder and fibrin glue. We then evaluated the structural, morphological, and mechanical properties of the scaffold and the in vitro release profile of ANG. Cell proliferation, viability, and adhesion were evaluated using endothelial cells *in vitro*, and angiogenesis and new bone formation were evaluated using a rabbit calvarial defect model *in vivo*.

**Results:**

Micro-computed tomography imaging showed that the bone powder was uniformly distributed in the scaffold, and scanning electron microscopy showed that the bone powder was bridged by polymerized fibrin. The porosity and compressive strength of the scaffolds were ~60 % and ~0.9 MPa, respectively, and were not significantly altered by ANG loading. *In vitro*, at 7 days, approximately 0.4 μg and 1.3 μg of the ANG were released from the FB/ANG 0.5 and FB/ANG 2.0, respectively and sustained slow release was observed until 25 days. The released ANG stimulated cell proliferation and adherence and was not cytotoxic. Furthermore, *in vivo* implantation resulted in enhanced angiogenesis, and new bone formation depended on the amount of loaded ANG.

**Conclusions:**

These studies demonstrate that a fibrin and bone powder scaffold loaded with ANG might be useful to promote bone regeneration by enhanced angiogenesis.

## Background

Bone reconstruction requires bone graft surgery in various conditions such as tumors, trauma, disease, and fracture of the bone. Although small defects self-regenerate, large bone defects remain challenging for bone replacement because they do not heal themselves. To overcome these problems, tissue engineering approaches have been applied to reconstruct the bone. In tissue engineering, biomaterial scaffolds are considered a crucial component because they provide a physical environment for bone formation and play an important role in cell growth, adhesion, and differentiation [1]. When scaffolds are used for bone regeneration, they often contain osteogenic growth factors or cytokines such as bone morphogenetic protein-2 (BMP-2) to enhance bone repair [2].

In addition to osteogenic stimulation, angiogenesis has recently been the focus of efforts to improve the clinical success of bone tissue repair. Angiogenesis is a key event in bone repair because new blood vessels serve as a route for providing oxygen, nutrients, and migration of bone precursor cells to the injury site [3]. Therefore, in bone tissue engineering, several studies have applied angiogenic growth factors such as vascular endothelial growth factor (VEGF) [4] and fibroblast growth factor (FGF) [5] to increase angiogenesis for bone repair.

Angiogenin (ANG) is a potent growth factor that can stimulate new blood vessel formation. ANG is a normal constituent of circulating blood with angiogenic activity [6]. It has also been found to be involved in neovascularization induced by various other angiogenic proteins such as FGF and VEGF [7].

Allogeneic mineralized bone powder has been widely used in the clinic to reconstruct bone defects because the molecular structure of bone is the same across species, making it possible to use bone from animal sources for bone grafts to enhance bone healing efficiency [8]. Several polymers such as a collagen [9], gelatin [10], chitosan [11], and fibrin [12] are used to fabricate porous scaffolds using bone powder. In particular, polymerized fibrin fibrous structures provide a temporary matrix during the rebuilding and repair of tissues [13]. Some studies have demonstrated that composite biomaterials and fibrin glue exhibit biocompatibility and increased osteoconductivity when compared to the biomaterials applied alone [14, 15]. Furthermore, fibrin has also been used as a delivery system because it serves as a binding reservoir for several growth factors, and the growth factors contained in fibrin gels are released in a delayed manner because the gel slows their diffusion [16].

Therefore, in this study, we fabricated an ANG-containing porous scaffold using fibrin glue and bovine bone powder. The microstructural and mechanical properties of the scaffold and the release pattern of ANG were characterized. The biocompatibility of the scaffold was evaluated using human umbilical vein cells (HUVECs) *in vitro*, and the tissue response and ability to induce angiogenesis and bone formation *in vivo* were evaluated using a rabbit calvarial defect model.

## Methods

### Scaffold preparation

In the present study, calcium phosphate–coated bovine bone powder (Biocera®; Oscotec, Chunan, Korea) was used as the bone powder material, and fibrin glue from a Greenplast kit was used (Greenplast kit; Green Cross Corp., Seoul, Korea). To construct the scaffolds, 30 mg of bone powder was placed in each hexahedron-shaped hole of a mold (8 mm × 8 mm × 1.5 mm). Next, 0.2 mL of fibrinogen solution (40 mg/mL in PBS) was prepared. Recombinant human ANG (rhANG; MybioSource, San Diego, CA) (0.5 μg or 2.0 μg) was then added into the fibrinogen solution to prepare the ANG-containing scaffold. Then, the fibrinogen or fibrinogen/ANG solution was added to the bone powder and mixed well. Thrombin (Greenplast Kit; 5 U/mL) solution (0.1 mL) was added, and the composites were rapidly blended. The resulting polymerized mixture was freeze-dried for 3 days to obtain a fibrin/bone powder (FB) scaffold.

### Micro-computerized tomography analysis

To evaluate the entire scaffold structure, samples were scanned with an aluminum filter using micro-computerized tomography (Micro-CT; Sky-Scan 1172TM; Skyscan, Kontich, Belgium). Three-dimensional and trans-sectional images were obtained from reconstructed scanned data set using CT-analyzer software (Skyscan).

### Microstructural analysis

To observe the structure of the scaffold, fabricated scaffolds were sputter-coated with gold for 120 s under vacuum. Then, the scaffolds were observed using a scanning electron microscope (SEM; EM-30; Coxem, Daejeon, Korea).

### Porosity and compressive strength analysis

Porosity was measured using a mercury intrusion porosimeter (AutoPore IV9500, Oak Ridge, TN). Briefly, scaffolds were sealed in a penetrometer, weighed, and subjected to analysis [17]. To evaluate the mechanical properties of the scaffold, compressive strength was analyzed. The fabricated scaffolds were subjected to a compression test using an Instron model 4505 universal test machine (Instron, Canton, MA) by applying a load via a 1 N load cell at a crosshead speed of 0.5 mm/min under ambient conditions.

### Determination of ANG release from scaffolds *in vitro*

The kinetics of ANG release from the FB/ANG scaffold were determined. The fabricated scaffolds were immersed in 2 mL of phosphate-buffered saline (PBS, pH 7.4). The samples were incubated at 37 °C under continuous agitation. At various time points, the supernatant was collected and fresh buffer was replenished. The quantitative determination of ANG was performed using an enzyme-linked immunosorbent assay (ELISA) kit (R&D Systems, Minneapolis, MN). Then, the cumulative release of ANG was calculated and represented as a percentage of the total amount of ANG in the scaffold.

### Cell culture

To evaluate the biocompatibility of the scaffold, immortalized HUVECs (EA.Hy926) were purchased from American Type Culture Collection (ATCC, Manassas, VA). The cells were cultured in DMEM (Gibco-BRL, Gaithersburg, MD) containing 10 % fetal bovine serum (FBS) and 1 % antibiotics at 37 °C under 5 % CO_2_ and 100 % humidity.

### Cell proliferation assay and evaluation of cytotoxicity

CellTiter96® Aqueous One solution (Promega, Madison, Wi) was used to measure cell proliferation. The EA.Hy926 cells were seeded and cultured on the FB and FB/ANG scaffolds. At predetermined time points (1, 5, 10, or 15 days), 200 μL of MTS reagent was mixed with 500 μL of culture media and added to each well. After incubation for 2 h, the supernatant was taken, and its absorbance was measured at 490 nm using an ELISA reader (SpectraMAX M3; Molecular Devices, Sunnyvale, CA). In addition, the cytotoxicity of the fabricated scaffold was evaluated using a Live/Dead® Viability/Cytotoxicity staining kit (Molecular Probe, Eugene, OR). After 3 days of cultivation, the specimen was rinsed with PBS to remove the phenol red, and reagent solution was added. After incubation for 30 min in a CO_2_ incubator, the samples were observed using an inverted fluorescence microscope (DM IL LED Fluo; Leica Microsystems, Wetzlar, Germany).

### Cell adhesion observation

SEM was used to observe cell adhesion to the scaffolds. After 5 days of culture, the samples were fixed with 2.5 % glutaraldehyde, and post-fixation was performed with 0.1 % osmium tetroxide (OsO_4_; Sigma). The sample was then dehydrated with a graded ethanol series (50 %, 75 %, 95 %, 100 %, and 100 %). The samples were then sputter-coated with gold and observed by SEM (EM-30; Coxem).

### Animal experiments

All animal experiments were performed according to the guidelines of the Wonkwang University Institutional Animal Care and Use Committee (Wonkwang University IACUC; WKU11-31). In this study, 3-month-old New Zealand white rabbits weighing 2.5–3.0 kg were used. To generate calvarial defects, the rabbits were anesthetized, the calvarium was exposed through a skin incision, and circular calvarial defects were made using a trephine bur (8 mm diameter) [18]. The FB and FB/ANG scaffolds were then implanted in the induced calvarial defects. After 1, 2, 4, and 8 weeks, the animals were sacrificed, and the bone defect regions were dissected out from the host bone. The extracted bone tissue was then fixed with 4 % paraformaldehyde buffered with 0.1 M phosphate (pH 7.2) for 5 days before further experiments.

### Micro-computed tomography

To analyze the newly formed bone, the bone specimens were scanned using a micro-CT system (Sky-Scan 1172TM; Skyscan, Kontich, Belgium) at 60 kV and 167 μA. Then, the image data were reconstructed using CT-analyzer software (Skyscan), and a three-dimensional image was reconstructed. A cylindrical region of interest (ROI) was positioned over the defect site. The volume of the newly formed bone was measured by assigning a threshold, and the percentage of the bone volume (% BV) was calculated by dividing the volume of the newly formed bone by the total volume within the ROI.

### Histology

After micro-CT scanning, the samples were dehydrated in a graded alcohol series (80–100 %), decalcified in 8 % formic acid/8 % HCl, and embedded in paraffin. Sections with a thickness of 5 μm were prepared from the samples and mounted on slides, and the samples were then stained with hematoxylin-eosin and Goldner’s Masson trichrome (MT) stain.

### Statistical analysis

All experiments were performed in triplicate, and statistical analyses were performed using GraphPad Prism statistical analysis software (GraphPad Software, San Diego, CA). Significant differences among groups were identified by ANOVA followed by *t*-test. Values in the text are expressed as the means ± standard deviation (SD), and *P* < 0.05 was considered statistically significant.

## Results

### Structural characterization of FB and FB/ANG scaffolds

**Fig. 1:**
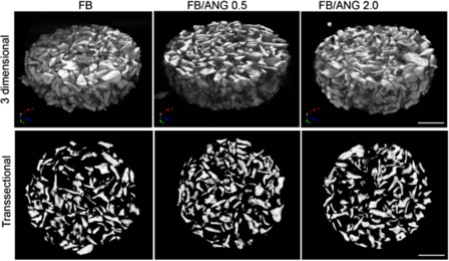
Representative micro-computed tomography images. Three-dimensional (**upper panel**) and trans-sectional images (**lower panel**) of fibrin glue/bone powder scaffold (FB), FB loaded with 0.5 μg of angiogenin (ANG) (FB/ANG 0.5), and FB loaded with 2.0 μg of ANG (FB/ANG 2.0) from micro-computed tomography (micro-CT) analysis. Scale bar = 2 mm

**Fig. 2:**
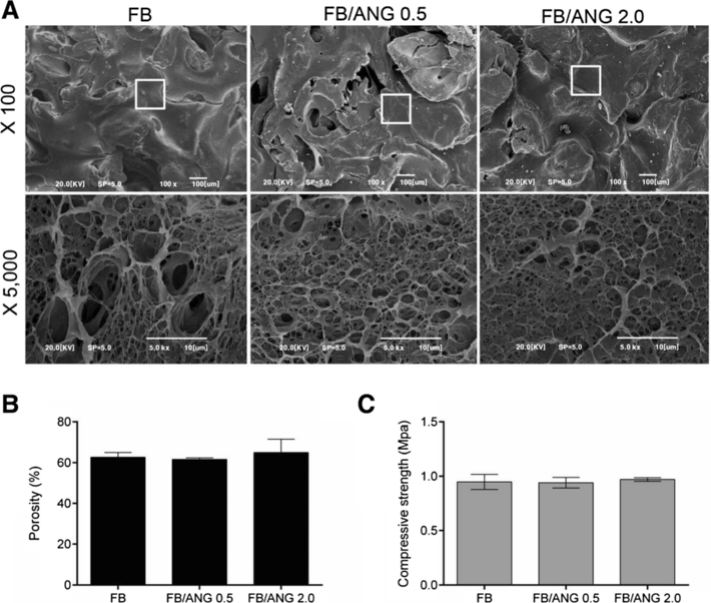
Characterization of fibrin glue/bone powder scaffold. Scanning electron microscopy (SEM) showed that the polymerized fibrin formed a layer and branched among the bone powder particles. In addition, the fibrin layer contained numerous micron-sized pores (**a**). The porosity (**b**) and compressive strength (**c**) were not affected by ANG loading in the scaffold

In this study, we successfully fabricated ANG-containing porous scaffolds using fibrin glue and bovine bone powder. Micro-CT imaging showed that the bovine bone powder was uniformly distributed in the FB, FB/ANG 0.5, and FB/ANG 2.0 scaffolds. In addition, trans-sectional micro-CT images indicated that numerous pores were present in all scaffolds (Fig. 1). SEM imaging of the fabricated scaffolds showed that the fibrin glue formed fibrin layers that branched among the bone powder particles. In addition, high-magnification observation showed that the fibrin layers consisted of a large number of micro-pores because of the formation of fibrin networks (Fig. 2a).

### Measurement of porosity and compressive strength

In bone tissue engineering, the porosity of the scaffold is important for angiogenesis, cell migration, and nutrient supplementation [19, 20]. Therefore, we assessed the porosity of the fabricated scaffolds. The porosity values of the FB, FB/ANG 0.5, and FB/ANG 2.0 scaffolds were 61.94 % ± 0.52 %, 61.61 % ± 0.73 %, and 61.56 % ± 1.07 %, respectively, which were not significantly different from each other (Fig. 2b). These results demonstrate that the porosity of the scaffold is not influenced by the addition of ANG. In addition, we measured the compressive strength of the scaffold because the mechanical properties are also important for bone reconstruction. The compressive strength values of the FB, FB/ANG 0.5, and FB/ANG 2.0 scaffolds were 0.94 ± 0.06 MPa, 0.95 ± 0.04 MPa, and 0.97 % ± 0.01 %, respectively, which were also not significantly different from each other (Fig. 2c).

### Release kinetics of ANG

**Fig. 3:**
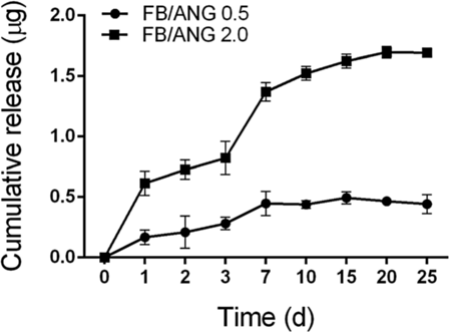
*In vitro* cumulative release of angiogenin (ANG) from scaffolds. The values represent the mean ± standard deviation (SD). At 7 days, approximately 0.4 μg and 1.3 μg of the ANG were released from the FB/ANG 0.5 and FB/ANG 2.0, respectively. Then the sustained slow release was observed until 25 days. The data shown are the mean ± SD

To evaluate ANG release kinetics from the scaffolds, ANG release over time was determined by ELISA (Fig. 3). Until the 7 days, fast release pattern were observed both of FB/ANG 0.5 and FB/ANG 2.0 scaffolds. Especially, at 7 days, approximately 0.4 μg and 1.3 μg of the ANG were released from the FB/ANG 0.5 and FB/ANG 2.0, respectively. And the slow release was sustained from 8 days to 25 days.

### Biocompatibility

**Fig. 4:**
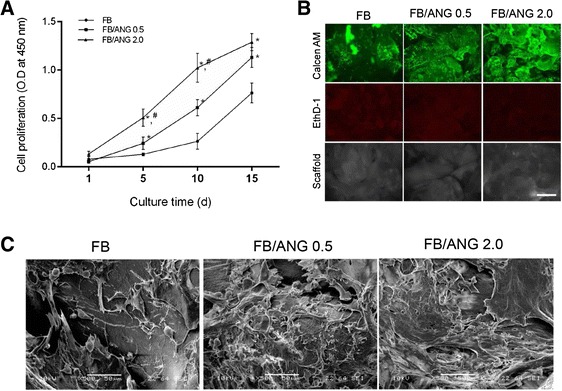
*In vitro* cellular biocompatibility of the scaffold. The biocompartibility was evaluated using an immortalized human umbilical vein cell line (EA.Hy926). Cells were cultured on each scaffold and proliferated gradually over time on all scaffolds. However, the proliferation was the highest on the FB scaffold loaded with 2 μg of ANG (FB/ANG 2.0) (**a**). After 3 days of cultivation, live/dead staining showed that most of the cells were viable (stained green by Calcein AM), whereas dead cells (stained red by EthD-1) were not observed. Scale bar = 500 μm (**b**). To evaluate cell adhesion, scanning electron microscopy (SEM) (C) was performed after 5 days of cultivation. SEM images showed that cell adhesion increased as ANG content increased (**c**). The data shown are the mean ± SD of three independent experiments. * and # indicate significantly different when compared with the FB scaffold and FB/ANG 0.5 scaffold, respectively

The effects of the addition of ANG on cell proliferation, cytotoxicity, and cell adhesion were evaluated. At day 1 of culture, the proliferation of cells cultured on the scaffolds was not significantly different among scaffold groups. However, with increased culture time, proliferation was significantly increased in cells cultured on the FB/ANG scaffold. In addition, the proliferation of cells on the FB/ANG 2.0 scaffold was significantly higher than that of cells on the FB/ANG 0.5 scaffold (Fig. 4a). Live/dead fluorescence imaging showed that most cells were viable on all scaffolds. Furthermore, the density of live green cells was consistent with the results of the MTS assay (Fig. 4b). To determine whether cells could attach to the scaffold, cells were cultured and their morphology observed by SEM after 5 days of culturing. SEM imaging showed that cells adhered and grew on the scaffolds. Attached cells were rarely observed on the FB scaffold but were more frequently observed on scaffolds constructed with increasing ANG concentrations (Fig. 4c). These results are also consistent with the findings of our MTS assay.

### *In vivo* analysis of neovascularization and new bone formation

**Fig. 5:**
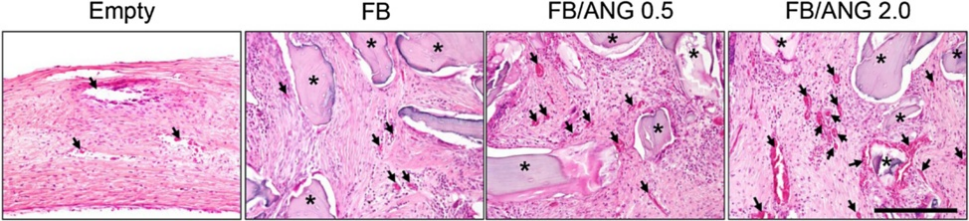
Hematoxylin-eosin-stained image of blood vessel formation in the central area of the calvarial defect site at 2 weeks after implantation. Many blood vessels clearly penetrated into the connective tissue and near the new bone in the FB/ANG 2.0 group. In addition, multinuclear cells and fibroblasts infiltrated into the connective tissue. Asterisk: residual material bone powder, black arrow: blood vessels. Empty: non-treated defect, FB: fibrin glue/bone scaffold alone, FB/ANG 0.5; FB scaffold loaded with 0.5 μg of ANG, FB/ANG 2.0; FB scaffold loaded with 2.0 μg of ANG. Scale bar = 250 μm

**Fig. 6:**
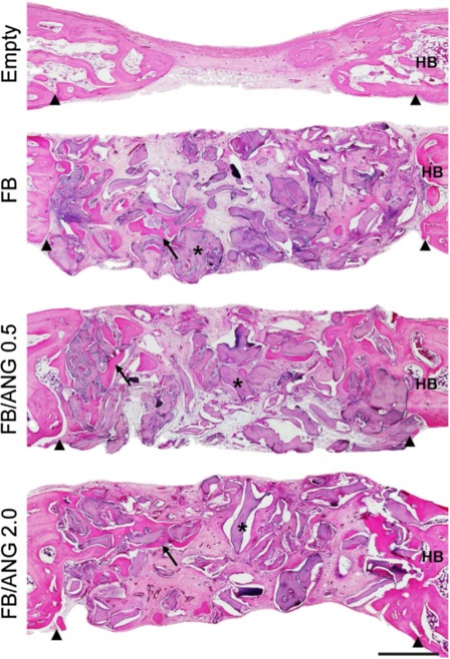
Microscopic evaluation of calvarial sections stained with hematoxylin-eosin after 8 weeks. Remaining bone particle material (asterisk) was observed in all scaffold-implanted groups. Newly generated mineralized bone (black arrow) was observed near the margin of the defect site (arrowhead) in the calvarial bone. HB represents the host bone. Scale bar = 500 μm

**Fig. 7:**
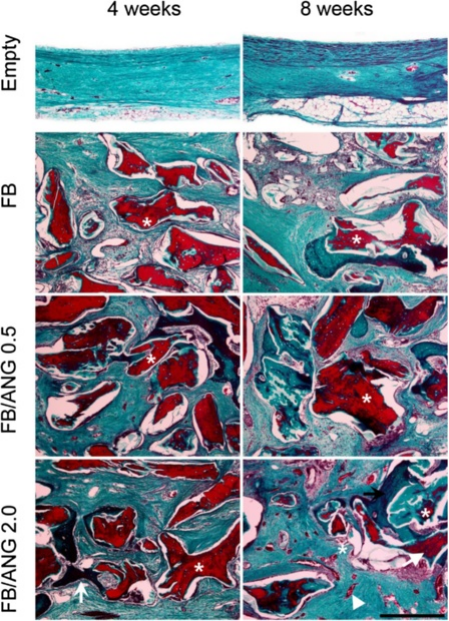
Goldner’s Masson trichrome staining of regenerated bone in the central area of the calvarial defect after implantation for 4 weeks and 8 weeks. After implantation for 4 weeks, mature bone (arrow) was distinctly observed around the residual material bone particles in the angiogenin-containing groups. Chronic inflammatory cells were also observed to infiltrate all defect areas. After implantation for 8 weeks, the greatest extent of regenerated bone (arrow) and blood vessels (arrowhead) was observed in the FB/ANG 2.0 group. Asterisk: residual material bone particles. Scale bar = 250 μm

To determine the effects of ANG-containing FB scaffolds on bone regeneration, we implanted the scaffolds in critical-sized defects in rabbits. At 2 weeks post-implantation, more blood vessels supplying connective tissue were clearly observed in the FB/ANG groups than in the FB scaffold group, and the number of blood vessels increased as the ANG concentration increased (Fig. 5). When characterizing bone regeneration at 8 weeks, new bone formation was observed around the margin of the defect site in the group with untreated defects. In contrast, new bone formation was observed not only around margin of the defective site but also around the bone powder in the defective site in the groups implanted with FB and FB/ANG scaffolds. Extensive newly formed bone was observed in the FB/ANG groups, and the bone had coalesced with the host bone. Furthermore, extensive new bone was observed in the group implanted with FB/ANG 2.0 scaffolds (Fig. 6). Images of the central area of the calvarial defects collected after MT staining showed large amounts of fibroblastic connective tissue in the group with untreated defects, whereas mature new bone was clearly observed in the FB and FB/ANG groups. In addition, more newly formed bone was observed in the FB/ANG 2.0 group than in the FB/ANG 0.5 group (Fig. 7).

### Micro-computed tomography evaluation

**Fig. 8:**
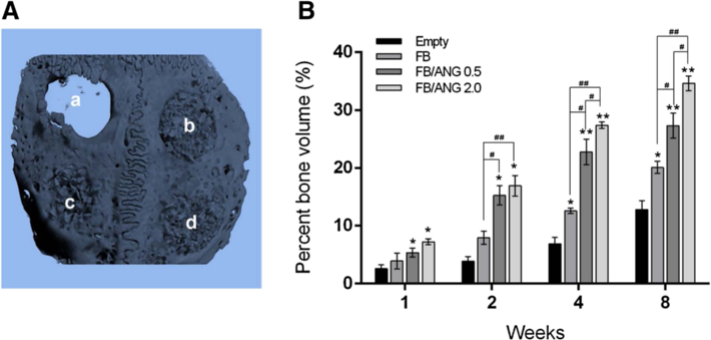
Three dimensional images of calvarial defects after implantation for 8 weeks (**a**). Newly regenerated bone covered the entire defect with scaffold treatment after implantation for 8 weeks. a; empty, b; FB, c; FB/ANG 0.5, d; FB/ANG 2.0. B. The % bone volume generated in the bone defects. The amount of new bone significantly increased over the study duration in the FB/ANG-implanted groups (**b**). Furthermore, the FB/ANG 2.0 group showed higher new bone formation than the FB/ANG 0.5 group. Each column value represents the mean ± SD. * and # indicate significantly different when compared with empty and FB scaffold groups, respectively (*n* = 4, *, #*P* < 0.05 and **, ##*P* < 0.01)

Reconstructed three-dimensional micro-CT images of non-implanted (empty) and implanted FB, FB/ANG 0.5, and FB/ANG 2.0 scaffolds are shown in Fig. 8a. In all groups, bone regeneration occurred from the margins of the defect site. In particular, new bone was generated not only in the margin of the defect but also in the central area of the defect among the bone powder in the bone powder-containing scaffolds (FB, FB/ANG 0.5, and FB/ANG 2.0). Quantification of bone regeneration revealed significant differences in the group with untreated defects and the groups implanted with FB and FB/ANG scaffolds. In particular, at 8 weeks, the newly formed bone volume was significantly higher in the FB/ANG 2.0 implanted group (34.67 % ± 1.25 %) than in the untreated defect (12.82 % ± 1.49 %), FB (20.12 % ± 1.08 %), and FB/ANG 0.5 (27.34 % ± 2.17 %) groups (Fig. 8b). The results of the micro-CT analysis were similar to those of the histological analysis.

## Discussion

Bone tissue engineering is promising for treating large bone defects in patients with severe bone loss. Therefore, many studies have attempted to improve bone tissue defect repair using biological scaffolds. One of the major factors in bone tissue engineering is angiogenesis [3]. Therefore, in scaffold design, porous structure is important because porosity enables cell infiltration, nutrient inflow, and vascular ingrowth [20]. Furthermore, current strategies for bone tissue regeneration focus on the combination of scaffolds and growth factors with osteoinductive and angiogenic potential to promote bone ingrowth [21]. Various studies have reported scaffold systems fabricated to deliver angiogenic growth factors such as VEGF and FGF [22, 23]. However, the application of ANG-containing scaffolds has not yet been evaluated in bone tissue engineering.

In this study, we attempted to prepare ANG-containing FB scaffolds for the first time, and vascularization and bone regeneration capacity were evaluated. Fibrinogen (40 mg/mL) and thrombin (5 U/mL) were used to prepare the scaffolds because these concentrations were optimized in pilot experiments [24]. Micro-CT imaging (Fig. 1) showed that the scaffolds were successfully fabricated with a uniform distribution of bone powder. Furthermore, SEM showed that the polymerized fibrin formed layers bound to the bone powder granules, resulting in the construction of a three-dimensional scaffold. When scaffolds are prepared using fibrin glue, their stiffness is influenced by the type and content of protein added, and the mechanical strength of fibrin matrix was shown to be increased by composition with collagen protein [25]. However, in the present study, there were no significant differences between FB scaffolds and FB/ANG scaffolds with regard to porosity and mechanical strength. These results suggest that ANG does not affect the porosity and mechanical properties of the FB scaffold, probably because of the low protein content and the nature of the protein, which may not affect the fibrin network.

During bone regeneration, angiogenesis precedes osteogenesis. Therefore, several studies have attempted the fabrication of angiogenic growth factor-releasing scaffolds [6, 7]. For instance, Leach et al. fabricated an angiogenic growth factor (VEGF)-coated scaffold and showed that it induced angiogenesis and accelerated bone healing in rat bone defects [26]. The VEGF release profile showed an initial rapid burst release pattern within approximately 5 days. Jeon et al. showed that the release profile of loaded FGF growth factor was influenced by the fibrinogen and thrombin concentration in a fibrin-based scaffold [27]; when the fibrin gel was prepared using fibrinogen at 94.3 mg/mL and thrombin at 33.3 U/mL, 60 % of the growth factor was released in the first 3 days. In contrast, our results showed that the initial burst release occurred in the first 7 days, with approximately 60 % of the ANG released, followed by a slow release profile that was sustained until 25 days. The different release profiles observed may be the result of different concentrations of fibrinogen and thrombin [27] and also differences in parameters such as morphological properties, material components, and preparation method [16].

In vitro cell culture is the starting point to evaluate the biocompatibility of developed scaffolds. In this study, cell proliferation and cytotoxicity were determined to evaluate biocompatibility. Several studies have reported that ANG stimulates endothelial cell proliferation and angiogenesis [7, 28]. In the present study, the MTS and cytotoxicity assays showed that FB/ANG enhanced cell proliferation and was not cytotoxic. Furthermore, cell adhesion was analyzed in SEM images (Fig. 4c), which showed that a higher amount of cells grew and adhered on FB/ANG scaffolds than on FB scaffolds. These results indicate that cell adhesion was also enhanced by addition of ANG to the scaffold because ANG supports the adhesion of endothelial cells [29].

Our in vitro results suggest that FB/ANG scaffolds could enable bone formation through enhanced angiogenesis. Therefore, in this study, we examined whether ANG-containing FB scaffolds could improve new bone formation using an *in vivo* rabbit calvarial defect model, which has been widely used to evaluate the performance of scaffolds in bone regeneration.

In bone tissue engineering, several growth factors (e.g., VEGF, FGF2, and PDGF) have been used to provide increased vascularization because new vessels support bone regeneration [3]. Comez et al. [23] reported enhanced bone healing in rat calvarial bone defects using FGF2 and a poly-l/d-lactide scaffold. Kaigler et al. reported enhanced bone regeneration using VEGF and poly (l/d-lactide and glycolide) scaffolds in irradiated rat osseous defects [30]. These previous studies indicate that angiogenic growth factors not only increase angiogenesis but also improve bone regeneration.

Given the pivotal role of ANG in angiogenesis, several studies have reported that neovascularization induced by ANG may enhance the healing of tissues such as the liver [31] and meniscus [32]. In addition, a previous study attempted the application of ANG with a collagen-chitosan scaffold for skin tissue engineering [33]. These previous reports suggest that ANG also can be used as a potential angiogenic growth factor in bone tissue engineering. In our *in vivo* study, the number of new blood vessels was significantly increased at 2 weeks in the group implanted with ANG-containing scaffolds. Furthermore, the regenerated bone volume was also significantly increased by ANG treatment. Although the exact mechanism was not evaluated in this study, these results suggest that induced vascularization by scaffold-released ANG improved new bone regeneration.

## Conclusions

In summary, we prepared an ANG-containing FB scaffold. The scaffold not only provided a suitable environment for the adhesion and proliferation of endothelial cells but also increased cell proliferation and adhesion. Furthermore, ANG-containing scaffolds promoted vascularization and improved new bone regeneration in calvarial defects *in vivo*. This study highlights the potential of using ANG-containing FB scaffolds for bone tissue engineering applications. However, further studies of the exact mechanism of angiogenesis and osteogenesis induced by FB/ANG are warranted.

## Availability of supporting data

The data sets supporting the results of this article are inclued within the article.
